# A prognostic scoring system for conversion surgery after trastuzumab-based chemotherapy for human epidermal growth factor receptor 2-positive advanced gastric cancer

**DOI:** 10.1007/s00595-022-02515-6

**Published:** 2022-05-11

**Authors:** Takaaki Arigami, Daisuke Matsushita, Keishi Okubo, Masataka Shimonosono, Ken Sasaki, Yusuke Tsuruda, Yoshiaki Kita, Kan Tanabe, Shinichiro Mori, Shigehiro Yanagita, Yoshikazu Uenosono, Akihiro Nakajo, Hiroshi Kurahara, Takao Ohtsuka

**Affiliations:** 1 Department of Onco-Biological Surgery, Kagoshima University Graduate School of Medical and Dental Sciences, 8-35-1 Sakuragaoka, Kagoshima, 890-8520 Japan; 2 Department of Digestive Surgery, Breast and Thyroid Surgery, Kagoshima University Graduate School of Medical and Dental Sciences, Kagoshima, Japan

**Keywords:** Gastric cancer, Conversion surgery, Prognosis, Trastuzumab, HER2

## Abstract

**Purpose:**

To investigate the clinical indications and prognostic significance of surgical interventions after chemotherapy using trastuzumab-containing regimens for patients with human epidermal growth factor receptor 2 (HER2)-positive advanced gastric cancer (AGC).

**Methods:**

A total of 146 patients with AGC who underwent chemotherapy were enrolled in this retrospective study. Tumors with an immunohistochemistry (IHC) score of 3 + or an IHC score of 2 + plus fluorescence in situ hybridization positivity were defined as HER2-positive AGC. We devised a scoring system for predicting prognosis associated with conversion surgery.

**Results:**

Thirty-three patients received trastuzumab-based chemotherapy for HER2-positive tumors. Multivariate analyses identified advanced age, peritoneal dissemination, histologically undifferentiated tumors, and tumor response of progressive disease as independent prognostic factors for a worse prognosis. Twelve patients with HER2-positive AGC underwent conversion surgery. The conversion surgery group of patients with HER2-positive AGC had a better prognosis than the chemotherapy-alone group. A prognostic scoring system based on age, peritoneal dissemination, and histological type was significantly correlated with the presence or absence of conversion surgery and the prognosis of patients with HER2-positive AGC.

**Conclusions:**

Our scoring system has the clinical potential to predict prognosis associated with conversion surgery after trastuzumab-containing chemotherapy for patients with HER2-positive AGC.

## Introduction

Gastric cancer is the fifth most common malignancy and the second-leading cause of cancer-related death worldwide [1, 2]. However, with the rapid development of chemotherapy, the prognosis of patients with unresectable advanced gastric cancer (AGC) has improved dramatically. In particular, the Trastuzumab for Gastric Cancer (ToGA) trial demonstrated the clinical efficacy of trastuzumab as a first-line regimen for patients with human epidermal growth factor receptor 2 (HER2)-positive unresectable advanced or recurrent gastric cancer [3]. Consequently, the clinical application of trastuzumab has greatly impacted the strategic management of patients on chemotherapy. The 2018 Japanese Gastric Cancer Treatment Guidelines are currently applied in clinics, and strongly recommend trastuzumab-based chemotherapy for patients with HER2-positive AGC [4].

In recent years, conversion surgery has attracted attention as a promising tool for improving the prognosis of responders with AGC after chemotherapy [5–7]. However, few studies have assessed the optimal timing of surgical interventions and the selection of candidates for conversion surgery. Therefore, the prognostic impact of conversion surgery and its clinical indication remain unclear in patients with HER-2-positive AGC who receive trastuzumab-based chemotherapy. A prognostic scoring system determined by pre-therapeutic factors that can be applied to selected candidates suitable for conversion surgery after chemotherapy would be valuable for the effective management of patients with HER2-positive AGC.

The purpose of the present study was to investigate tumor response, the presence or absence of conversion surgery, and the prognosis of patients with HER2-positive AGC. Furthermore, we propose a prognostic scoring system for selecting surgical candidates from responders after trastuzumab-based chemotherapy for HER2-positive AGC.

## Methods

### Patients

We reviewed a total of 146 patients with unresectable AGC, who underwent chemotherapy at Kagoshima University Hospital (Kagoshima, Japan) between September 2010 and December 2020. Patients with synchronous and metachronous cancer in other organs and those with disease recurrence were excluded from the present study. All patients underwent blood examinations, esophagogastroduodenoscopy, endoscopic ultrasonography, and computed tomography (CT) before receiving chemotherapy. Patients were categorized and staged based on the tumor–node–metastasis classification for gastric carcinoma established by the International Union Against Cancer [8].

Table 1 shows the clinicopathological features of the enrolled patients. The study included 94 men and 52 women with a mean age of 65.9 years (age range, 30–87 years). Among the 146 patients, 104, 28, 12, and 2 had metastases at 1, 2, 3, and 4 distant metastatic sites, respectively. Furthermore, 33 and 89 patients had liver metastasis and peritoneal dissemination, respectively. Differentiated and undifferentiated tumors were identified in 36 and 110 patients, respectively. This study was approved by the Ethics Committee of Kagoshima University (approval number: 200043).

**Table 1:** Clinicopathological factors of the patients (*n* = 146)

Factor	*n* (%)
Sex	
Male	94 (64.4)
Female	52 (35.6)
Mean age (range), years	65.9 (30–87)
Tumor location	
Whole/upper	91 (62.3)
Middle/lower	55 (37.7)
Macroscopic type	
Type 1	6 (4.1)
Type 2	14 (9.6)
Type 3	78 (53.4)
Type 4	47 (32.2)
Type 5	1 (0.7)
Depth of tumor invasion	
cT2	3 (2.1)
cT3	13 (8.9)
cT4	130 (89.0)
Lymph node metastasis	
cN0	23 (15.8)
cN1	24 (16.4)
cN2	43 (29.5)
cN3	56 (38.4)
Clinical stage	
IV	146 (100.0)
Number of distant metastatic sites	
1	104 (71.2)
2	28 (19.2)
3	12 (8.2)
4	2 (1.4)
Liver metastasis	
H0	113 (77.4)
H1	33 (22.6)
Peritoneal dissemination	
P0	57 (39.0)
P1	89 (61.0)
Histological type	
Differentiated	36 (24.7)
Undifferentiated	110 (75.3)
HER2 status	
IHC 0	46 (31.5)
IHC 1 +	37 (25.3)
IHC 2 + and FISH –	30 (20.5)
IHC 2 + and FISH +	14 (9.6)
IHC 3 +	19 (13.0)

*FISH* fluorescence in situ hybridization, *HER2* human epidermal growth factor receptor 2, *IHC* immunohistochemistry

### Immunohistochemistry and fluorescence in situ hybridization for the assessment of HER2 expression

The tumor biopsy specimens obtained prior to chemotherapy were subjected to immunohistochemistry (IHC) analysis. All paraffin-embedded specimens were cut into 4-µm-thick slices and transferred to a slide. IHC was conducted using the Hercept test kit (Dako, Carpinteria, CA) based on the protocol recommended by the manufacturer. According to the Hercept test scoring criteria, staining intensity was scored as 0, 1 + , 2 + , or 3 + [3].

Fluorescence in situ hybridization (FISH) was conducted using the Abbott PathVysion HER2 DNA Probe Kit protocol (Abbott Laboratories, Abbott Park, Des Plaines, IL) according to the manufacturer’s instructions. HER2 gene amplification was assessed by calculating the number of HER2 and centromere enumerator probe 17 (CEP 17) signals in 20 adjacent interphase tumor cell nuclei, which were examined under a fluorescent microscope with the appropriate filters. A positive HER2 gene amplification status was defined as a HER2:CEP17 ratio of ≥ 2.0 [3].

In the present study, patients who had tumors with an IHC score of 3 + or an IHC score of 2 + plus FISH positivity were defined as having HER2-positive gastric cancer [3].

### Assessment of tumor response to chemotherapy and histological response

Tumor response to chemotherapy was assessed once every three cycles and was determined based on the Response Evaluation Criteria in Solid Tumors (RECIST) [9]. This study grouped tumor response into the following two categories: progressive disease (PD) and non-PD.

Residual tumor status and the histological response at the primary tumor site were classified according to the Japanese classification of gastric carcinoma [10]. Accordingly, the surgical status for the assessment of residual tumors was categorized into R0 (no residual tumor), R1 (microscopic residual tumor), and R2 (macroscopic residual tumor). The histological tumor response was grouped into grades 0 (no effect), 1a (very slight effect), 1b (slight effect), 2 (considerable effect), and 3 (complete response).

### Clinical indication for conversion surgery after chemotherapy

Conversion surgery after chemotherapy was clinically indicated for patients with a performance status (PS) of 0–2 and a tumor status predicted to achieve R0 curative resection. The decision about suitability for surgical treatment was also based on the patient’s health condition and the physician’s discretion.

### Statistical analysis

The relationship between the score obtained using the prognostic scoring system and the presence or absence of conversion surgery was assessed using the chi-squared test. Survival time was defined as the duration from chemotherapy initiation to death or last follow-up. Kaplan–Meier survival curves were generated, and prognostic differences were assessed using the log-rank test. Prognostic factors were evaluated using univariate and multivariate analyses (Cox proportional hazards regression modeling). All data were analyzed using JMP14 (SAS Institute Inc., Cary, NC, USA). A *p* value of < 0.05 was considered significant.

## Results

### Characteristics of the patients with HER2-positive AGC

Among the 146 patients enrolled, the IHC scores denoting HER2 status of 46, 37, 30, 14, and 19 patients were as follows: 0, 1 + , 2 + plus FISH negativity, 2 + plus FISH positivity, and 3 + , respectively (Table 1). Accordingly, 33 patients (22.6%) had HER2-positive tumors.

Table 2 summarizes the clinicopathological factors of the 33 patients (age range 37–82 years; mean age 66.1 years) with HER2-positive AGC. Among the 33 patients, 3 and 30 had type 4 and type non-4 tumors, respectively. Moreover, 10 and 11 patients had liver metastasis and peritoneal dissemination, respectively. Staging laparoscopy before chemotherapy was done for 12 of 33 patients with HER2-positive AGC. Five patients had positive peritoneal cytology (CY1). The histological type of tumor was differentiated in 13 patients and undifferentiated in 20 patients. All patients received trastuzumab-based chemotherapy.

**Table 2:** Clinicopathological features of the patients with human epidermal growth factor receptor 2-positive advanced gastric cancer (*n* = 33)

Factor	*n* (%)
Sex	
Male	27 (81.8)
Female	6 (18.2)
Mean age (range), years	66.1 (37–82)
Tumor location	
Whole	4 (12.1)
Upper	17 (51.5)
Middle	4 (12.1)
Lower	8 (24.2)
Macroscopic type	
Type 1	3 (9.1)
Type 2	3 (9.1)
Type 3	24 (72.7)
Type 4	3 (9.1)
Depth of tumor invasion	
cT2	3 (9.1)
cT3	5 (15.2)
cT4	25 (75.8)
Lymph node metastasis	
cN0	3 (9.1)
cN1	5 (15.2)
cN2	7 (21.2)
cN3	18 (54.5)
Clinical stage	
IV	33 (100.0)
Number of distant metastatic sites	
1	23 (69.7)
2	7 (21.2)
3	3 (9.1)
Liver metastasis	
H0	23 (69.7)
H1	10 (30.3)
Peritoneal dissemination	
P0	22 (66.7)
P1	11 (33.3)
Peritoneal lavage cytology	
CY0	7 (21.2)
CY1	5 (15.2)
CYX	21 (63.6)
Histological type	
Differentiated	13 (39.4)
Undifferentiated	20 (60.6)
First-line regimen of trastuzumab-based chemotherapy	
Platinum	28 (84.8)
Taxane	5 (15.2)

*AGC* advanced gastric cancer, *CY p*eritoneal lavage cytology, *HER2* human epidermal growth factor receptor 2

### Tumor response to trastuzumab-based chemotherapy and prognosis

**Fig. 1:**
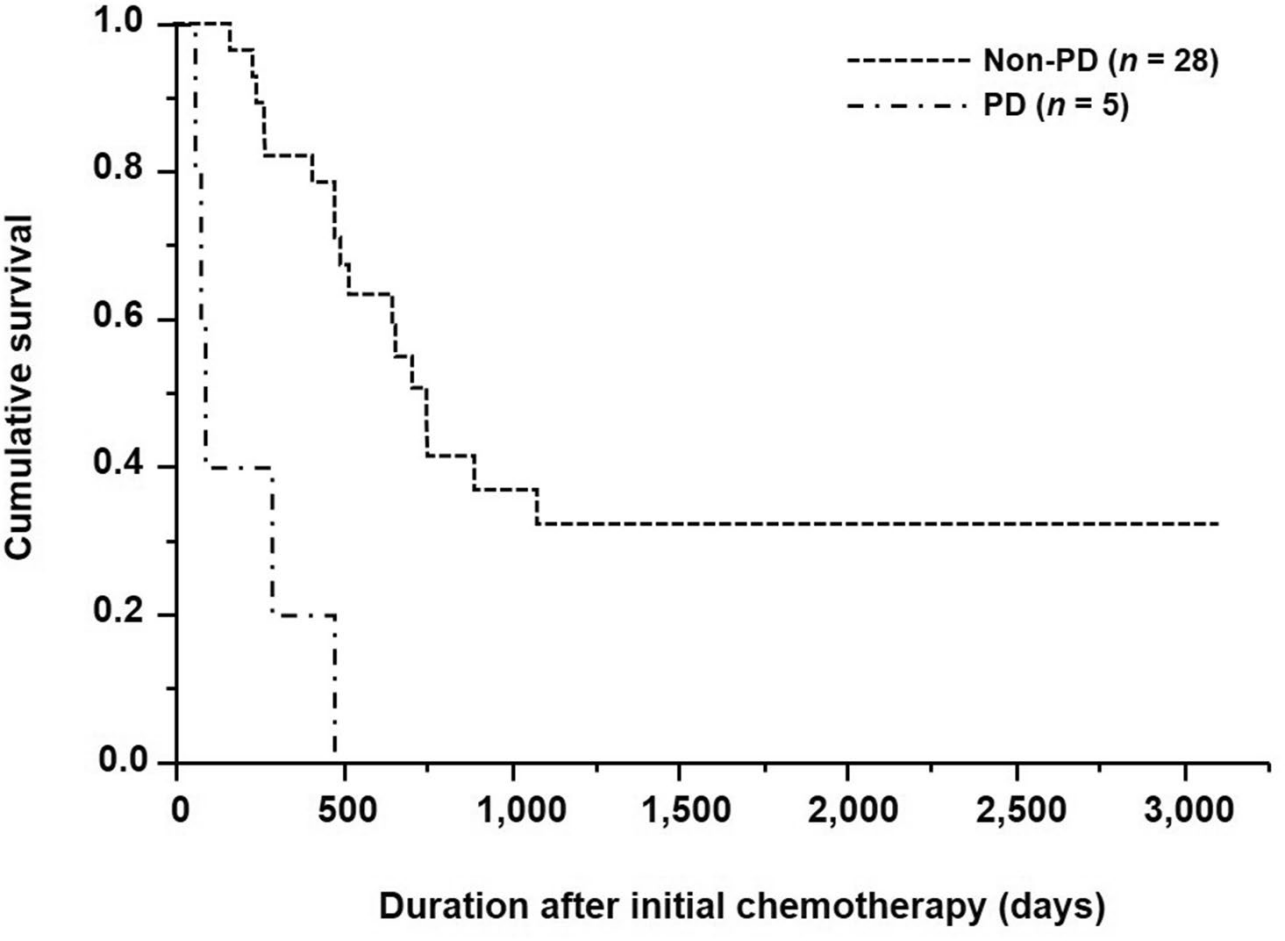
Kaplan–Meier survival curves based on tumor response for patients with HER2-positive advanced gastric cancer. *PD* progressive disease

Among the 33 patients with HER2-positive AGC who received trastuzumab-based chemotherapy, 5 and 28 showed PD and non-PD, respectively, as per RECIST. Therefore, the disease control rate was 84.8% (28/33). Patients with HER2-positive AGC who showed PD and non-PD had a median survival time (MST) of 90 and 754 days, respectively (Fig. 1). The survival differences based on tumor responses were significant for patients with HER2-positive AGC (*p* < 0.0001).

### Univariate and multivariate analyses of survival in patients with HER2-positive AGC

For univariate and multivariate analyses, the cutoff value of age was set at 70 years, based on the median age. Univariate analysis of patients with HER2-positive AGC identified that age (< 70 vs. ≥ 70 years), macroscopic type (type non-4 vs. type 4), peritoneal dissemination (P0 vs. P1), histological type (differentiated vs. undifferentiated), and tumor response (non-PD vs. PD) were significantly related to survival (*p* = 0.0207, *p* = 0.0116, *p* = 0.0117, *p* = 0.0020, and *p* = 0.0002, respectively; Table 3). Multivariate analysis revealed that age, peritoneal dissemination, histological type, and tumor response were independent prognostic factors (*p* = 0.0028, *p* = 0.0321, *p* = 0.0026, and *p* = 0.0033, respectively; Table 3).

**Table 3:** Univariate and multivariate analyses of survival for patients with human epidermal growth factor receptor 2-positive advanced gastric cancer (*n* = 33)

Independent factor	Univariate analysis			Multivariate analysis		
	Hazard ratio	95% CI	*p* value	Hazard ratio	95% CI	*p* value
Sex			0.5879			
Female	1.000	Reference				
Male	1.352	0.454–4.021				
Age			0.0207			0.0028
< 70 years	1.000	Reference		1.000	Reference	
≥ 70 years	2.778	1.169–6.601		4.893	1.730–13.840	
Tumor location			0.3085			
Middle/lower	1.000	Reference				
Whole/upper	1.596	0.649–3.925				
Macroscopic type			0.0116			0.0820
Type non-4	1.000	Reference		1.000	Reference	
Type 4	5.241	1.447–18.979		3.715	0.847–16.302	
Depth of tumor invasion			0.0814			
cT2–3	1.000	Reference				
cT4	2.960	0.873–10.033				
Lymph node metastasis			0.7692			
cN0–2	1.000	Reference				
cN3	1.136	0.485–2.662				
Number of distant metastatic sites			0.9763			
1	1.000	Reference				
≥ 2	1.014	0.412–2.496				
Liver metastasis			0.0725			
H0	1.000	Reference				
H1	2.228	0.930–5.340				
Peritoneal dissemination			0.0117			0.0321
P0	1.000	Reference		1.000	Reference	
P1	3.577	1.328–9.633		3.525	1.114–11.156	
Histological type			0.0020			0.0026
Differentiated	1.000	Reference		1.000	Reference	
Undifferentiated	7.044	2.048–24.226		8.012	2.070–31.011	
HER2 status			0.4337			
IHC 2 + and FISH +	1.000	Reference				
IHC 3 +	0.709	0.300–1.677				
First-line regimen of trastuzumab-based chemotherapy			0.4411			
Platinum	1.000	Reference				
Taxane	0.619	0.182–2.100				
Tumor response to chemotherapy			0.0002			0.0033
Non-PD	1.000	Reference		1.000	Reference	
PD	9.990	2.980–33.488		7.140	1.926–26.464	

*AGC* advanced gastric cancer, *CI* confidence interval, *FISH* fluorescence in situ hybridization, *HER2* human epidermal growth factor receptor 2, *IHC* immunohistochemistry, *PD* progressive disease

### Conversion surgery after chemotherapy and pathological findings in patients with HER2-positive AGC

Among the 33 patients with HER2-positive AGC, 12 (36.4%) underwent conversion surgery after chemotherapy. The median number of courses of trastuzumab-based chemotherapy before conversion surgery was 5 (3–17). Table 4 details the surgical procedures and pathological findings. Proximal gastrectomy, distal gastrectomy, total gastrectomy, and esophagectomy were performed in three (25.0%), four (33.3%), four (33.3%), and one (8.3%) patients, respectively. Furthermore, one (8.3%), four (33.3%), three (25.0%), and four (33.3%) patients underwent D1, D1 + , D2, and D2 + lymphadenectomy, respectively. Four (33.3%), three (25.0%), two (16.7%), and three (25.0%) patients had pathological T1, T2, T3, and T4 tumors, respectively. Moreover, seven (58.3%), two (16.7%), two (16.7%), and one (8.3%) patients had pathological N0, N1, N2, and N3 stage disease, respectively. R0 curative resection was achieved in all patients (*n* = 12). The histological tumor response analysis revealed that 10 and 2 patients had grade 1a and 1b disease, respectively. Among the 12 patients who underwent conversion surgery, 10 (83.3%) received adjuvant chemotherapy using S-1.

**Table 4:** Surgical procedures and pathological findings in patients with human epidermal growth factor receptor 2-positive advanced gastric cancer (*n* = 12)

Factor	*n* (%)
Surgical procedure	
Proximal gastrectomy	3 (25.0)
Distal gastrectomy	4 (33.3)
Total gastrectomy	4 (33.3)
Esophagectomy	1 (8.3)
Lymph node dissection	
D1	1 (8.3)
D1 +	4 (33.3)
D2	3 (25.0)
D2 +	4 (33.3)
Depth of tumor invasion	
pT1	4 (33.3)
pT2	3 (25.0)
pT3	2 (16.7)
pT4	3 (25.0)
Lymph node metastasis	
pN0	7 (58.3)
pN1	2 (16.7)
pN2	2 (16.7)
pN3	1 (8.3)
Residual tumor status	
R0	12 (100.0)
Histological response	
Grade 1a	10 (83.3)
Grade 1b	2 (16.7)

*AGC* advanced gastric cancer

**Fig. 2:**
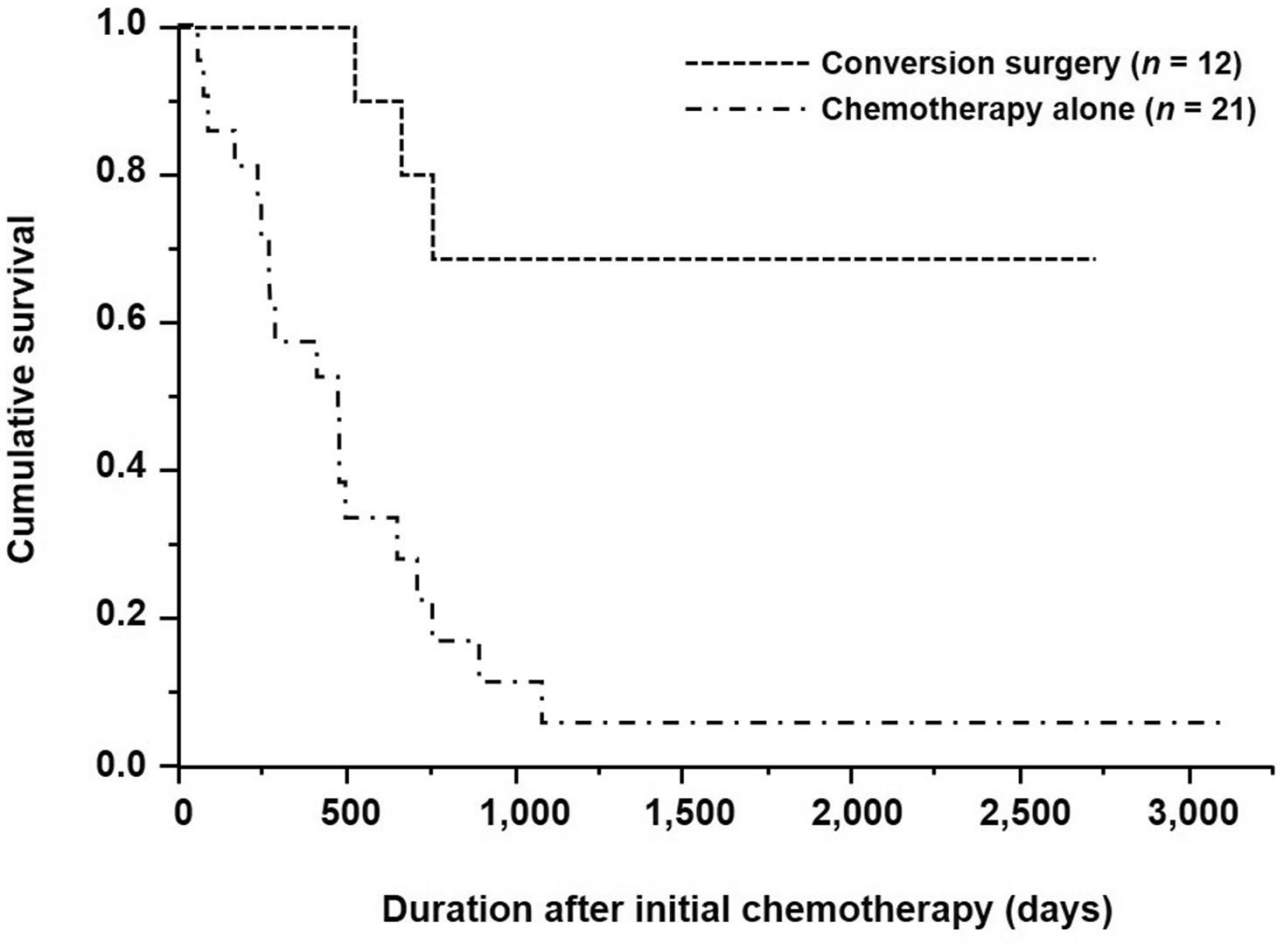
Kaplan–Meier survival curves based on the presence or absence of conversion surgery for patients with HER2-positive advanced gastric cancer

**Fig. 3:**
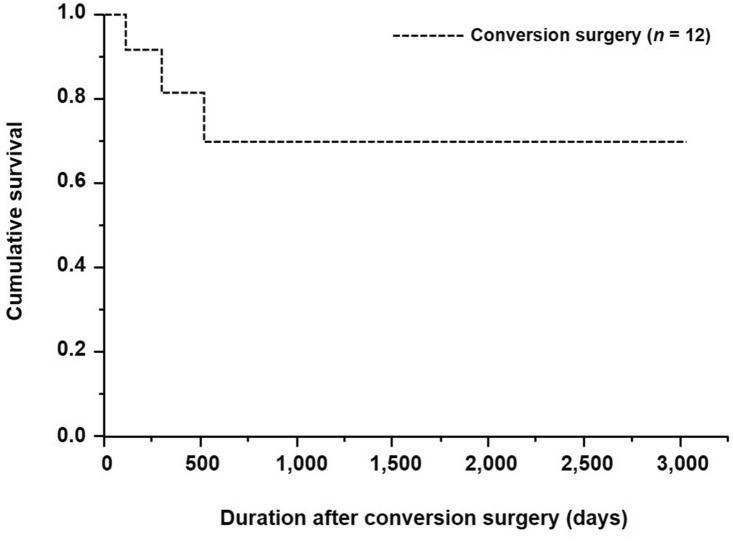
Kaplan–Meier survival curves after conversion surgery for patients who underwent conversion surgery

Table 5 shows the relationship between the presence or absence of conversion surgery and clinicopathological factors. Conversion surgery was significantly associated with age, the depth of tumor invasion, and histological type (*p* = 0.0374, *p* = 0.0152, and *p* = 0.0265, respectively). The 5-year overall survival (OS) rates of patients with HER2-positive AGC who underwent conversion surgery vs. those who received chemotherapy alone were 68.6% and 5.6%, respectively (*p* = 0.0002; Fig. 2). Furthermore, the 5-year OS rates after conversion surgery was 69.8% for the patients who underwent conversion surgery (Fig. 3).

**Table 5:** Relationship between the presence or absence of conversion surgery and clinicopathological factors (*n* = 33)

Factor	Treatments, *n* (%)		*p* value
	Conversion surgery group	Chemotherapy alone group	
	(*n* = 12)	(*n* = 21)	
Gender			1.0000
Female	2 (16.7)	4 (19.0)	
Male	10 (83.3)	17 (81.0)	
Mean age, years	60.5 ± 12.1	69.3 ± 11.9	0.0374
Performance status			0.0648
0–2	12 (100.0)	15 (71.4)	
3–4	0 (0.0)	6 (28.6)	
Tumor location			0.7161
Middle/lower	5 (41.7)	7 (33.3)	
Whole/upper	7 (58.3)	14 (66.7)	
Macroscopic type			0.2841
Type non-4	0 (0.0)	3 (14.3)	
Type 4	12 (100.0)	18 (85.7)	
Depth of tumor invasion			0.0152
cT2–3	6 (50.0)	2 (9.5)	
cT4	6 (50.0)	19 (90.5)	
Lymph node metastasis			0.3005
cN0–2	7 (58.3)	8 (38.1)	
cN3	5 (41.7)	13 (61.9)	
Number of distant metastatic sites			0.7098
1	9 (75.0)	14 (66.7)	
≥ 2	3 (25.0)	7 (33.3)	
Liver metastasis			0.2593
H0	10 (83.3)	13 (61.9)	
H1	2 (16.7)	8 (38.1)	
Peritoneal dissemination			0.1389
P0	10 (83.3)	11 (55.0)	
P1	2 (16.7)	9 (45.0)	
Histological type			0.0265
Differentiated	8 (66.7)	5 (23.8)	
Undifferentiated	4 (33.3)	16 (76.2)	
First-line regimen of trastuzumab-based chemotherapy			0.3275
Platinum	9 (75.0)	19 (90.5)	
Taxane	3 (25.0)	2 (9.5)	
Tumor response to chemotherapy			0.1329
Non-PD	12 (100.0)	16 (76.2)	
PD	0 (0.0)	5 (23.8)	

*PD* progressive disease

### Prognostic scoring system to predict suitability of clinical candidates for conversion surgery among patients with HER2-positive AGC

We established a prognostic scoring system to pre-therapeutically predict the suitability of candidates for conversion surgery after trastuzumab-based chemotherapy. This system consisted of three pre-therapeutic factors based on multivariate analysis of prognosis. Each factor was given a score of 1 point. The three factors were as follows: ≥ 70 years, the presence of peritoneal dissemination (P1), and undifferentiated tumor type. Accordingly, because the total score ranged from 0 to 3 points, patients were categorized into four groups.

**Fig. 4:**
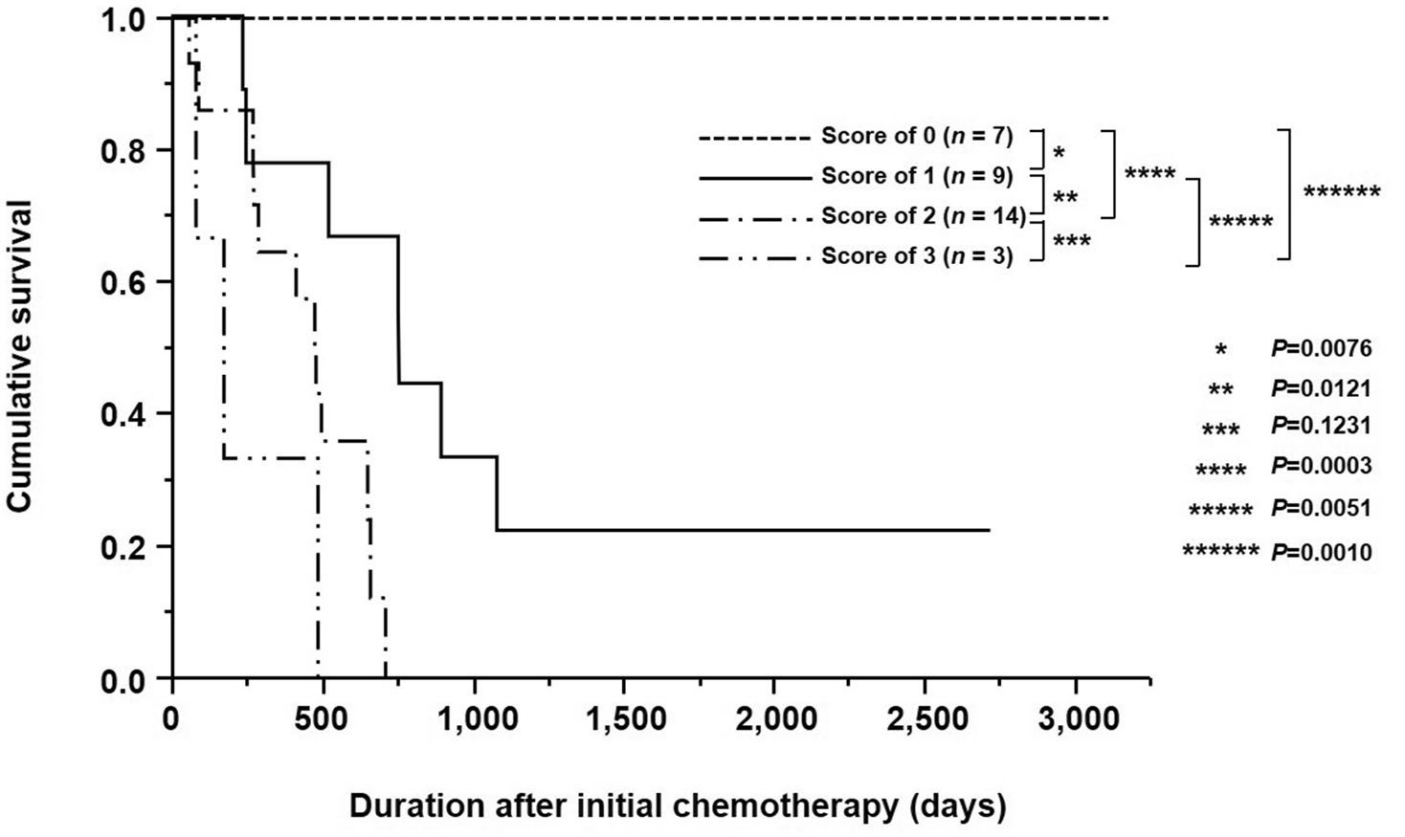
Kaplan–Meier survival curves based on the prognostic scoring system for patients with HER2-positive advanced gastric cancer

This scoring system revealed 7 (21.2%), 9 (27.3%), 14 (42.4%), and 3 (9.1%) patients with prognostic scores of 0, 1, 2, and 3 points, respectively. The prognostic score correlated significantly with the presence or absence of conversion surgery (*p* = 0.0065; Table 6). All patients who scored 0 points were alive at the time of writing this article (Fig. 4). Patients with scores of 1, 2, and 3 points had MSTs of 757, 478, and 169 days, respectively. There were significant prognostic differences among patients with each score, except for patients with scores of 2 vs. 3 (*p* < 0.05; Fig. 4).

**Table 6:** Relationship between the prognostic score and the presence or absence of conversion surgery

	Prognostic score (%)				*p* value
	0 (*n* = 7)	1 (*n* = 9)	2 (*n* = 14)	3 (*n* = 3)	
Conversion surgery					0.0065
Absence	1 (14.3)	5 (55.6)	12 (85.7)	3 (100.0)	
Presence	6 (85.7)	4 (44.4)	2 (14.3)	0 (0.0)

## Discussion

Currently, trastuzumab-based chemotherapy is recommended as a first-line treatment for patients with HER2-positive AGC [4]. However, the incidence of HER2 gene amplification and/or protein overexpression in patients with AGC has been reported to range from 7 to 34% [11–14]. The low HER2 positivity rate among patients with AGC implies that trastuzumab-based chemotherapy may have limited benefit for AGC. Therefore, we must establish if conversion surgery after trastuzumab-based chemotherapy is a promising tool for improving the prognosis of patients with HER2-positive AGC. To our knowledge, this is the first study to develop and evaluate a prognostic scoring system for selecting suitable candidates for conversion surgery from among patients with HER2-positive AGC being treated with trastuzumab-based chemotherapy.

In the clinical management of patients with HER2-positive AGC, changes in HER2 status during trastuzumab-based chemotherapy have been highlighted in relation to drug resistance to subsequent anti-HER2 chemotherapy. Seo et al. [15] enrolled 48 patients with HER2-positive AGC treated with trastuzumab-containing first-line chemotherapy and investigated HER2 expression retrospectively, using IHC and/or FISH in tumor specimens at baseline and after PD to chemotherapy. They reported that tumor specimens from 14 patients (29.2%) who showed PD response (disease progression) to trastuzumab-based chemotherapy, exhibited HER2-loss status [15]. Moreover, patients with a stable HER2 status had a response rate of 44% and a median progression-free survival (PFS) of 2.7 months, whereas those with HER2-loss conversion were non-responsive and had a shorter PFS [15]. Similarly, Saeki et al. [16] assessed HER2 status retrospectively in re-biopsied specimens obtained from patients with HER2-postive AGC after a PD response to trastuzumab-containing first-line chemotherapy. They found that the incidence of HER2-loss status was 60.6% in that patient population (20/33). These results may suggest the limitation of trastuzumab-based chemotherapy alone in the clinical management of patients with HER2-positive AGC. Collectively, the above findings imply that therapeutic strategies including surgical interventions may improve the prognosis of patients with HER2-positive AGC.

Recent studies have demonstrated the prognostic utility of conversion surgery after chemotherapy in patients with stage IV AGC [5–7, 17–19]. Beom et al. [17] reviewed clinicopathological factors and prognosis retrospectively in 101 patients with stage IV AGC (unknown HER2 status), who were treated with systemic chemotherapy followed by gastrectomy, and reported that the median OS time was 26.0 months. Our study indicated that the median OS time was not reached in the conversion surgery group of patients with HER2-positive AGC. Surprisingly, in the present study, the 5-year OS rate in the conversion surgery group with HER2-positive AGC was 68.6%. Hayano et al. [18] examined prognostic data retrospectively in six patients with HER2-positive AGC, who underwent conversion surgery after trastuzumab-containing chemotherapy, and found that the 3-year survival rate was 66.7%. These findings imply that patients with HER2-positive AGC may be more suitable candidates for conversion surgery after trastuzumab-based chemotherapy, with greater improvements in prognosis, than those with HER2-negative AGC.

In this study, one patient underwent esophagectomy with lymphadenectomy (Table 4). The patient had a type 2 tumor of the esophagogastric junction and CT indicated swelling of the upper thoracic paraesophageal lymph node (station no. 105). Tubular adenocarcinoma with HER2 score of 3 + was identified by pathological examination of station no. 105 obtained by endoscopic ultrasound guided fine needle aspiration and a clinical diagnosis of Siewert type II esophagogastric junction adenocarcinoma of HER2-positive (T2N0M1) was made. The patient showed a complete response after three courses of trastuzumab-based chemotherapy and underwent conversion surgery. Pathological examination revealed that the primary tumor invaded the mucosa (ypT1a), indicating histological grade 1a. No tumor cells were seen in the dissected lymph nodes including station no. 105 (ypN0). Accordingly, a pathological diagnosis of stage IA (ypT1aN0M0) was made and the patient received oral S-1 as adjuvant chemotherapy. There were no signs of disease recurrence 12 months postoperatively.

The clinical indication for conversion surgery in patients with HER2-positive AGC has not been defined or documented in the literature. Therefore, we devised a prognostic scoring system based on multivariate analysis of survival to identify suitable candidates. Age (< 70 vs. ≥ 70 years), peritoneal dissemination (P0 vs. P1), and histological type (differentiated vs. undifferentiated) were selected as important factors in our scoring system. These three pre-therapeutic factors are all well-established prognostic markers in patients with AGC [20–22]. However, none of the previous studies have assessed age, peritoneal dissemination, and histological type simultaneously as potential indicators for conversion surgery in patients with HER2-positive AGC after treatment with trastuzumab-based chemotherapy. This study is unique because we developed a prognostic scoring system based on three clinicopathological factors to establish the therapeutic strategy, including conversion surgery after trastuzumab-based chemotherapy, for patients with HER2-positive AGC.

The present study demonstrated a close relationship between our prognostic score and the presence or absence of conversion surgery. Among the seven patients with a score of 0, six (85.7%) underwent conversion surgery, whereas among the three patients with a score of 3, none underwent conversion surgery. Consequently, this prognostic scoring system may have clinical utility to predict the suitability of conversion surgery in the pre-therapeutic management of patients with HER2-positive AGC. Furthermore, our prognostic score correlated significantly with OS. In particular, all patients (*n* = 7) with a score of 0 were alive at the time of writing this article. On the other hand, patients with a score of 3 had worse prognosis than those with scores of 0, 1, or 2. These results suggest that this prognostic scoring system may be useful for predicting the prognosis associated with conversion surgery after trastuzumab-based chemotherapy for patients with HER2-positive AGC.

The present study has several limitations. First, it was a retrospective study conducted at a single institution, and the sample size was small (HER2-positive AGC patients: *n* = 33). Second, conversion surgery after chemotherapy was clinically indicated for patients with a PS of 0–2 and a tumor status predicted to achieve R0 curative resection based on their general condition. The treatment decision also depended on the physician’s decision. Moreover, one patient from the chemotherapy-alone group, with a complete response to trastuzumab-based chemotherapy survived for more than 5 years. Although we recommended conversion surgery, the patient declined. These limitations might have resulted in biases that could have impacted the results. Therefore, further large prospective studies are warranted to validate our findings.

In conclusion, a grading system based on age, peritoneal dissemination, and histological type has the clinical potential to predict the suitability of conversion surgery and prognosis for patients with HER2-positive AGC treated with trastuzumab-containing chemotherapy. We anticipate that our prognostic scoring system will serve as a promising tool for planning an aggressive strategy in the clinical management of patients with HER2-positive AGC.
